# Supplementary material for: HRD effects on first-line adjuvant chemotherapy and PARPi maintenance therapy in Chinese ovarian cancer patients
Source: NPJ Precis Oncol. 2023 May 31;7:51. doi: 10.1038/s41698-023-00402-y (PMC10232447; doi:10.1038/s41698-023-00402-y)
Supplement: Supplementary file 1 — Supplementary Information [file 41698_2023_402_MOESM1_ESM.pdf]

Supplementary Information

Supplementary Figures

- Supplementary Figure 1. The overview of patient cohorts included in this study
- Supplementary Figure 2. Association between HRR gene alterations and HRD score in the FACT cohort
- Supplementary Figure 3. Association between *BRCA1* promoter methylation and HRD score in the FACT cohort
- Supplementary Figure 4. The patient survival analysis stratified by different causes of HRD in the FACT cohort
- Supplementary Figure 5. The platinum sensitivity status stratified by HRD status in the FACT cohort
- Supplementary Figure 6. Association between TP53 alteration and HRD status in *BRCA1/2* wild-type participants
- Supplementary Figure 7. The patient survival analysis stratified by TP53 BILOF

Supplementary Tables

- Supplementary Table 1. DDR genes and HRR gene lists
- Supplementary Table 2. Baseline clinical characteristics in the FACT cohort
- Supplementary Table 3. Baseline clinical characteristics in the FPMT cohort

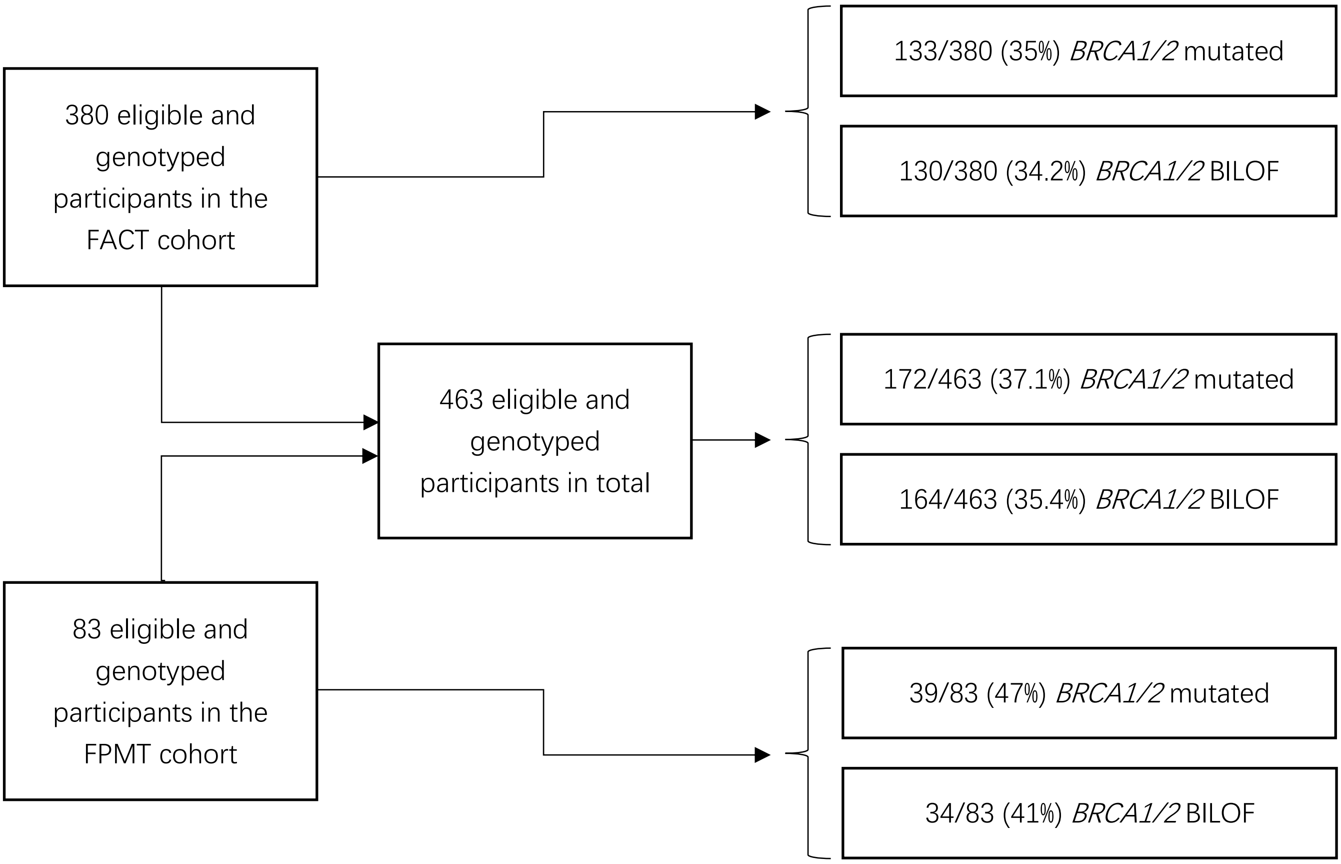

Supplementary Figure 1. The overview of patient cohorts included in this study

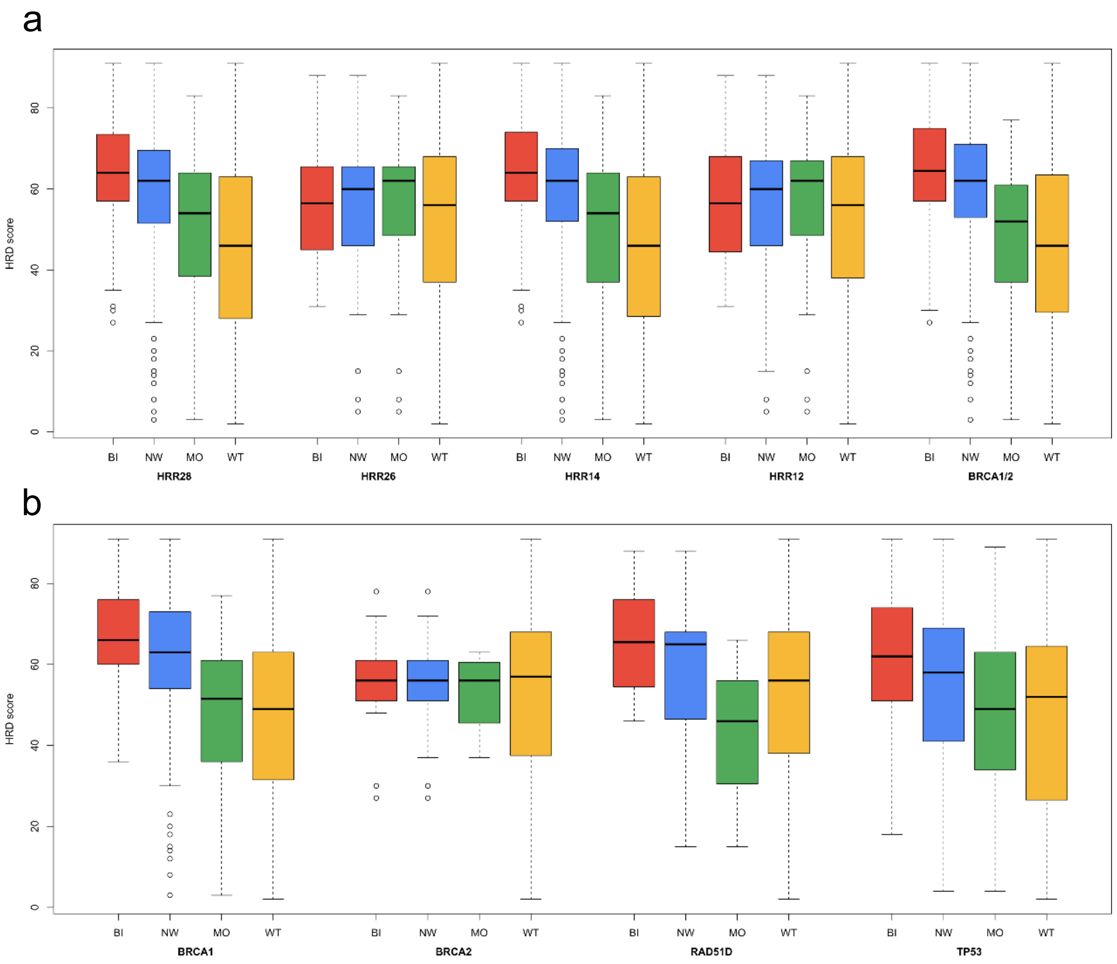

Supplementary Figure 2. Association between HRR gene alterations and HRD score in the FACT cohort

(a, b) Boxplots illustrate the distribution of HRD scores stratified by alteration type of (a) HRR genes and (b) selective DDR genes. On each boxplot, the lower whisker, lower bound of box, center line, upper bound of box, and upper whisker represent the minimum, lower quartile, median, upper quartile, and maximum, respectively. Alteration types include BI, MO, NW, and WT. BI, bi-allelic loss-of-function; MO, mono-allelic loss-of-function; NW, non-wild-type; and WT, wild-type.

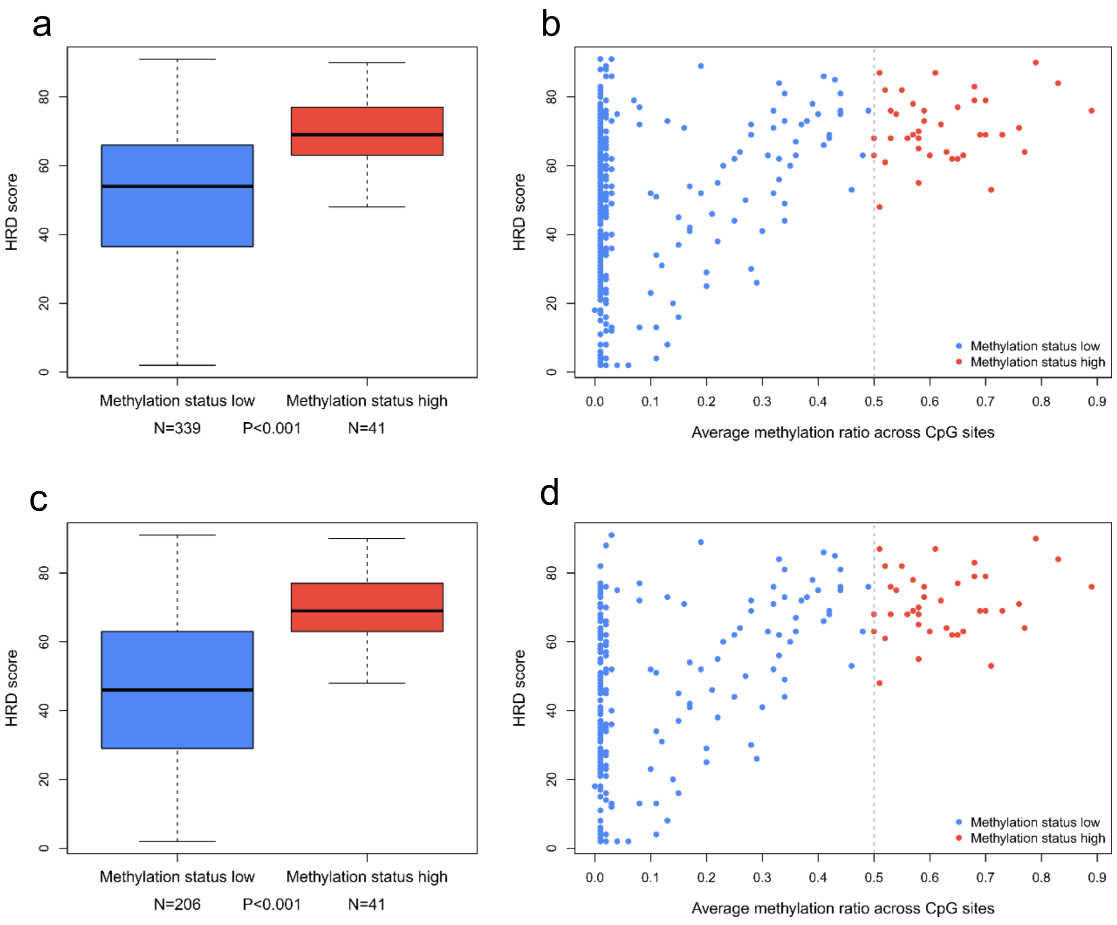

Supplementary Figure 3. Association between *BRCA1* promoter methylation and HRD score in the FACT cohort

Boxplots illustrate the distribution of HRD scores stratified by *BRCA1* promoter methylation status (*BRCA1* promoter methylation score ≥ 0.5 or not) in (a) the FACT cohort and (c) *BRCA1/2* wild-type participants in the FACT cohort. Scatter plots illustrate the relationship between *BRCA1* promoter methylation score and HRD score in (b) the FACT cohort and (d) *BRCA1/2* wild-type participants in the FACT cohort. P-values were calculated by the Wilcoxon test. On each boxplot, the lower whisker, lower bound of box, center line, upper bound of box, and upper whisker represent the minimum, lower quartile, median, upper quartile, and maximum, respectively.

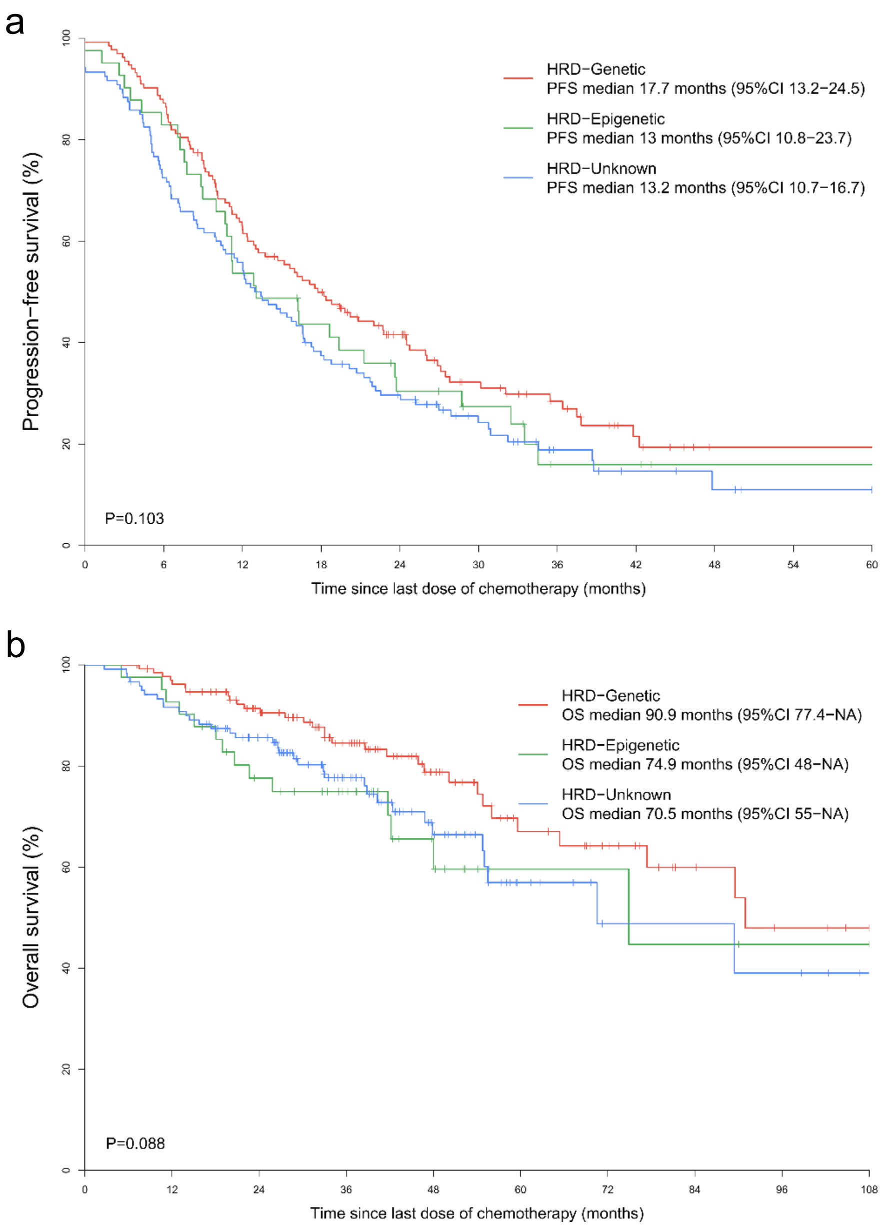

Supplementary Figure 4. The patient survival analysis stratified by different causes of HRD in the FACT cohort

(a) PFS and (b) OS analyses stratified by different causes of HRD in HRD-positive participants in the FACT cohort. HRD-Genetic represents HRD-positive status caused by genetic changes (*BRCA1/2* mutated); HRD-Epigenetic represents HRD-positive status caused by epigenetic changes (*BRCA1/2* wild-type, and *BRCA1* promoter methylation status high); HRD-Unknown represents HRD-positive status caused by unknown reasons (*BRCA1/2* wild-type, *BRCA1* promoter methylation status low, and HRD status positive). P-values were calculated by a log-rank test.

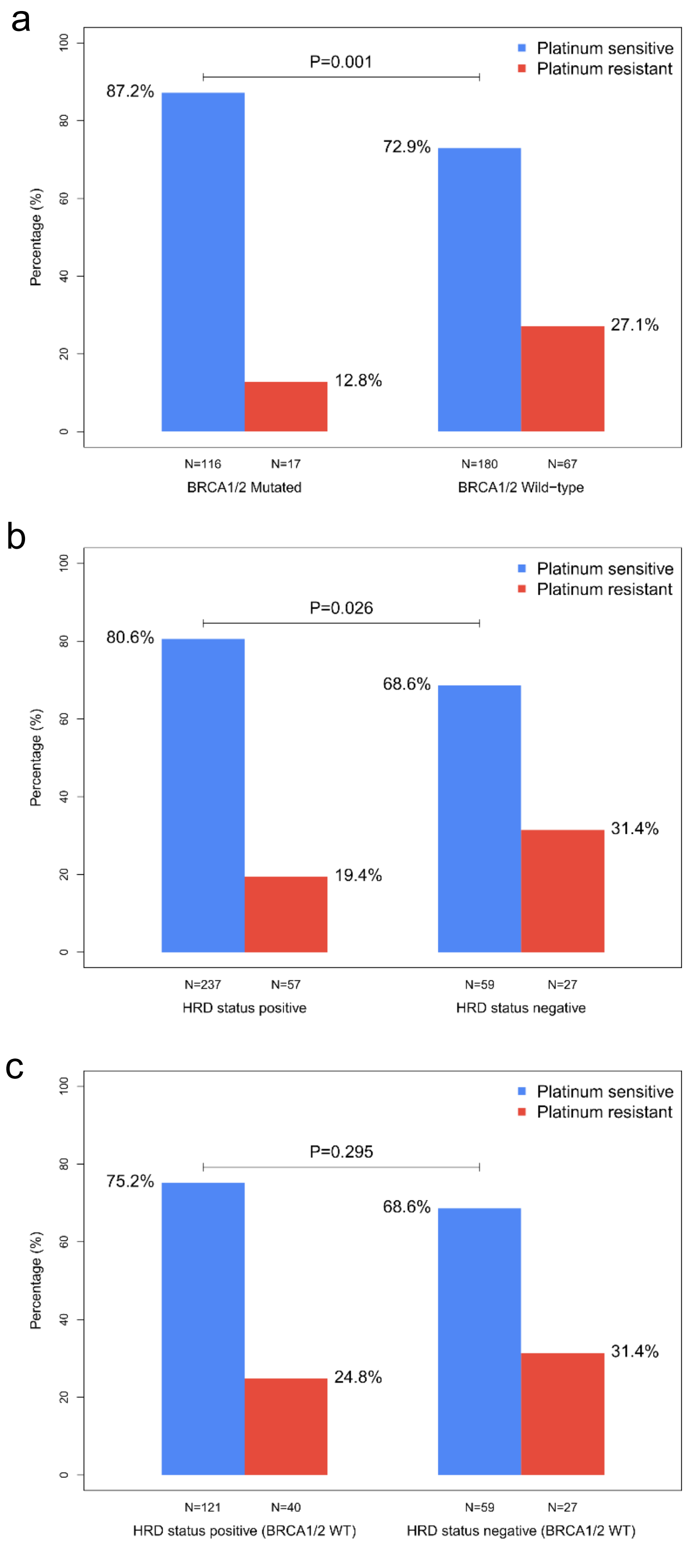

Supplementary Figure 5. The platinum sensitivity status stratified by HRD status in the FACT cohort

(a-c) PSS analyses in the FACT cohort stratified by (a) *BRCA1/2* mutation, (b) HRD status, and (c) HRD status in *BRCA1/2* wild-type participants. WT, wild-type.

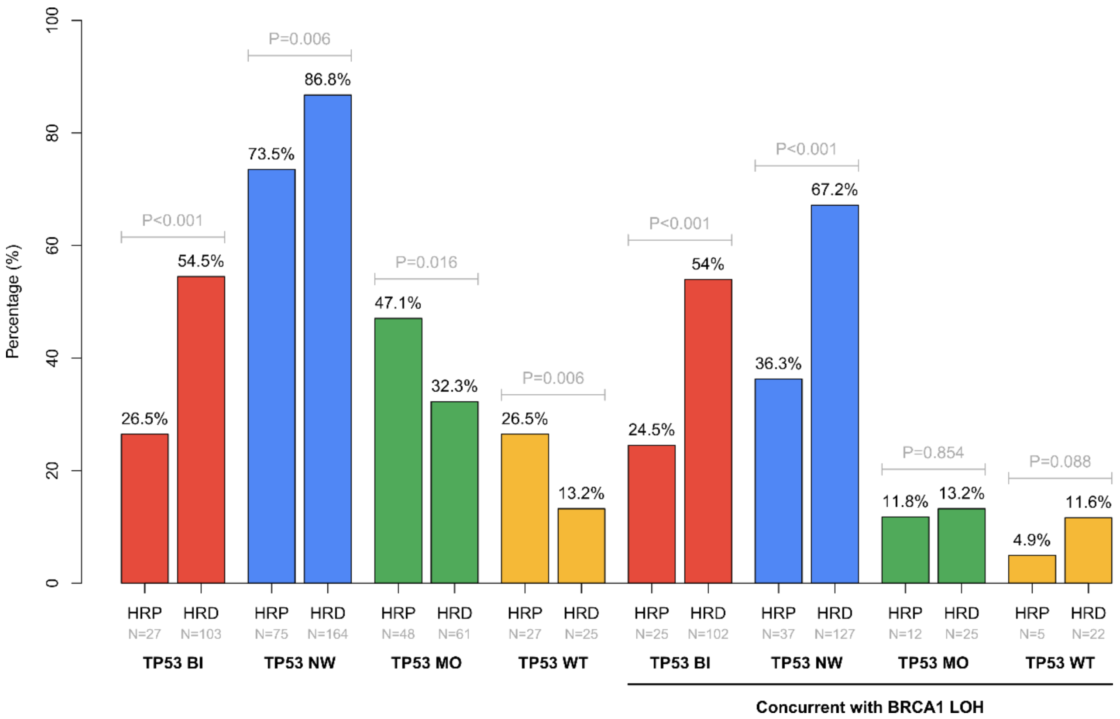

Supplementary Figure 6. Association between TP53 alteration and HRD status in *BRCA1/2* wild-type participants

Each bar illustrates the percentage of a specific *TP53* alteration type out of the participants who were HRP (HRD-negative) or HRD (HRD-positive). Alteration types include BI, MO, NW, and WT. BI, bi-allelic loss-of-function; MO, mono-allelic loss-of-function; NW, non-wild-type; WT, wild-type. On the right side is the percentages of different *TP53* alteration types concurrent with *BRCA1* LOH. P-values were calculated by Fisher's exact test.

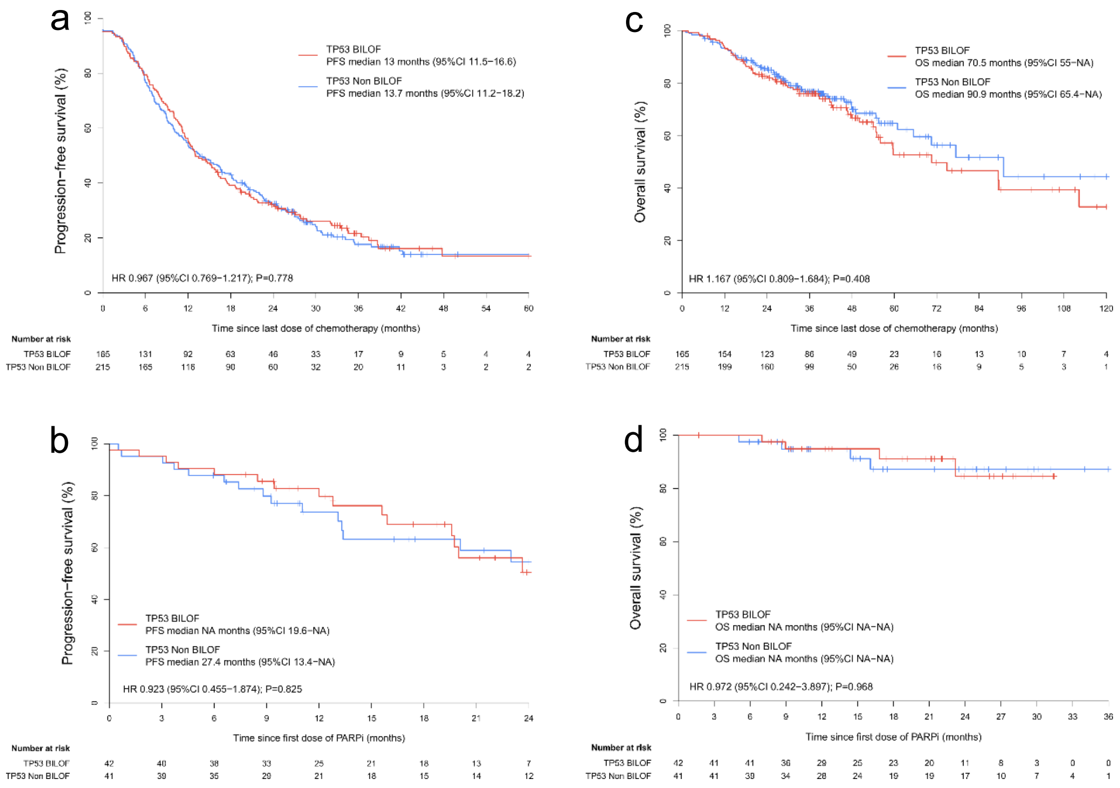

Supplementary Figure 7. The patient survival analysis stratified by TP53 BILOF (a, b) PFS analyses stratified by *TP53* BILOF in (a) the FACT cohort and (b) the FPMT cohorts. (c, d) OS analyses stratified by *TP53* BILOF in (c) the FACT cohort and (d) the FPMT cohorts.

Supplementary Table 1. DDR genes and HRR gene lists

DDR[2] genes	HRR gene lists[1]			
	**HRR28**	**HRR26**	**HRR14**	**HRR12**
ATM	√	√	√	√
ATR	√	√		
BAP1	√	√		
BARD1	√	√	√	√
BRCA1	√		√	
BRCA2	√		√	
BRIP1	√	√	√	√
CDH1				
CDK12	√	√	√	√
CHEK1	√	√	√	√
CHEK2	√	√	√	√
EMSY[3]	√	√		
EPCAM				
FAM175A	√	√		
FANCA	√	√		
FANCC	√	√		
FANCD2	√	√		
FANCI	√	√		
FANCL	√	√	√	√
MLH1				
MRE11A	√	√		
MSH2				
MSH6				
NBN	√	√		
PALB2	√	√	√	√
PMS2				
PPP2R2A	√	√		
PTEN	√	√		
RAD50	√	√		
RAD51B	√	√	√	√
RAD51C	√	√	√	√
RAD51D	√	√	√	√
RAD54B	√	√		
RAD54L	√	√	√	√
STK11				
TP53				

[1]If at least one gene in a gene list is mutated, then the gene list is defined as mutated.

[2]Most genes listed here are involved in HRR and mismatch repair pathways, except for *STK11*, *TP53*, and *CDH1*.

[3]Amplification and overexpression of *EMSY*, as an alternative way by which tumors selectively inactivate the BRCA pathway (Bell et al., 2011), indicate its mechanism for HRD is different from the "loss of function" of other HRR genes. However, *EMSY* amplification analysis was not performed in this study.

Supplementary Table 2. Baseline clinical characteristics in the FACT cohort

Characteristic	HRD status negative (N=86)	HRD status positive (N=294)
Age, years	54 (48-62)	53 (47-61)
Cancer type		
Fallopian tube cancer	8 (9.3)	34 (11.6)
Ovarian cancer	78 (90.7)	258 (87.8)
Primary peritoneal cancer	0 (0)	2 (0.7)
Histological type		
Grade-3 endometroid	3 (3.5)	5 (1.7)
High-grade serous	83 (96.5)	289 (98.3)
FIGO Stage		
II	7 (8.1)	26 (8.8)
III	69 (80.2)	233 (79.3)
IV	10 (11.6)	35 (11.9)
Surgery type		
IDS	50 (58.1)	138 (46.9)
PDS	36 (41.9)	156 (53.1)
Surgery residual[1]		
R0	50 (58.1)	183 (62.2)
R1	28 (32.6)	79 (26.9)
R2	8 (9.3)	32 (10.9)
Concurrent use of bevacizumab[2]		
Without	80 (93)	271 (92.2)
With	6 (7)	23 (7.8)
Round of chemotherapy[3]	8 (6-9)	8 (6-9)
Pre-treatment CA125[4], U/ml	674.55 (262-1549.5)	705.95 (248.95-1442.08)

Note: Data are median (IQR) or n (%).

Abbreviations: FIGO, International Federation of Gynecology and Obstetrics; PDS, primary debulking surgery; IDS, interval debulking surgery.

[1]R0, no residual; R1, residual < 1cm; R2, residual ≥ 1cm.

[2]with concurrent use of bevacizumab is defined as having received at least one dose of bevacizumab during first-line treatment.

[3]Round of chemotherapy is the sum of round of first-line neoadjuvant chemotherapy and round of first-line adjuvant chemotherapy.

[4]Pre-treatment CA125 is the CA125 level measured before any form of first-line treatment is administered, including surgery and chemotherapy.

Supplementary Table 3. Baseline clinical characteristics in the FPMT cohort

Characteristic	HRD status negative (N=16)	HRD status positive (N=67)
Age, years	56 (50-71)	52 (46-58)
Cancer type		
Fallopian tube cancer	0 (0)	1 (1.5)
Ovarian cancer	15 (93.8)	66 (98.5)
Primary peritoneal cancer	1 (6.2)	0 (0)
Histological type		
Grade-3 endometroid	1 (6.2)	0 (0)
High-grade serous	15 (93.8)	67 (100)
FIGO Stage		
II	1 (6.2)	5 (7.5)
III	10 (62.5)	48 (71.6)
IV	5 (31.2)	14 (20.9)
Surgery type		
IDS	11 (68.8)	36 (53.7)
PDS	5 (31.2)	31 (46.3)
Surgery residual[1]		
R0	10 (62.5)	52 (77.6)
R1	3 (18.8)	8 (11.9)
R2	3 (18.8)	7 (10.4)
PARPi type		
Niraparib	12 (75)	21 (31.3)
Olaparib	4 (25)	46 (68.7)
Concurrent use of bevacizumab[2]		
Without	10 (62.5)	53 (79.1)
With	6 (37.5)	14 (20.9)
Round of chemotherapy[3]	6 (6-7)	6 (6-7)
Pre-treatment CA125[4], U/ml	502.05 (198.2-844.25)	799 (263.74-1657.77)
Time to PARPi[5], days	37 (31-58)	46 (37-57)

Note: Data are median (IQR) or n (%).

Abbreviations: FIGO, International Federation of Gynecology and Obstetrics; PDS, primary debulking surgery; IDS, interval debulking surgery.

[1]R0, no residual; R1, residual < 1cm; R2, residual ≥ 1cm.

[2]With concurrent use of bevacizumab is defined as having received at least one dose of bevacizumab during first-line treatment.

[3]Round of chemotherapy is the sum of rounds of first-line neoadjuvant chemotherapy and rounds of first-line adjuvant chemotherapy.

[4]Pre-treatment CA125 is the CA125 level measured before any form of first-line treatment is administered, including surgery and chemotherapy.

[5]Time to PARPi stands is defined as the time from the last dose of first-line adjuvant chemotherapy to the first dose of first-line PARPi maintenance therapy.
