# Supplementary material for: HRD effects on first-line adjuvant chemotherapy and PARPi maintenance therapy in Chinese ovarian cancer patients
Source: NPJ Precis Oncol. 2023 May 31;7:51. doi: 10.1038/s41698-023-00402-y (PMC10232447; doi:10.1038/s41698-023-00402-y)
Supplement: Supplementary file 2 — Reporting Summary [file 41698_2023_402_MOESM2_ESM.pdf]

Corresponding author(s): Han Liang
Last updated by author(s): May 8 2023

Reporting Summary

Nature Portfolio wishes to improve the reproducibility of the work that we publish. This form provides structure for consistency and transparency in reporting. For further information on Nature Portfolio policies, see our Editorial Policies and the Editorial Policy Checklist.

Statistics

For all statistical analyses, confirm that the following items are present in the figure legend, table legend, main text, or Methods section.

n/a	Confirmed	
☐	☒	The exact sample size (*n*) for each experimental group/condition, given as a discrete number and unit of measurement
☐	☒	A statement on whether measurements were taken from distinct samples or whether the same sample was measured repeatedly
☐	☒	The statistical test(s) used AND whether they are one- or two-sided<br>*Only common tests should be described solely by name; describe more complex techniques in the Methods section.*
☐	☒	A description of all covariates tested
☐	☒	A description of any assumptions or corrections, such as tests of normality and adjustment for multiple comparisons
☐	☒	A full description of the statistical parameters including central tendency (e.g. means) or other basic estimates (e.g. regression coefficient) AND variation (e.g. standard deviation) or associated estimates of uncertainty (e.g. confidence intervals)
☐	☒	For null hypothesis testing, the test statistic (e.g. *F*, *t*, *r*) with confidence intervals, effect sizes, degrees of freedom and *P* value noted<br>*Give P values as exact values whenever suitable.*
☒	☐	For Bayesian analysis, information on the choice of priors and Markov chain Monte Carlo settings
☒	☐	For hierarchical and complex designs, identification of the appropriate level for tests and full reporting of outcomes
☐	☒	Estimates of effect sizes (e.g. Cohen's *d*, Pearson's *r*), indicating how they were calculated

Our web collection on statistics for biologists contains articles on many of the points above.

Software and code

Policy information about availability of computer code

Data collection	A custom bioinformatic analysis pipeline was used to detect single nucleotide variants (SNVs) and small insertions and deletion (indels) mutations in protein-coding regions and intron/exon boundaries of the 36 genes.
Data analysis	Statistical analyses were conducted using R 4.2.1.

For manuscripts utilizing custom algorithms or software that are central to the research but not yet described in published literature, software must be made available to editors and reviewers. We strongly encourage code deposition in a community repository (e.g. GitHub). See the Nature Portfolio guidelines for submitting code & software for further information.

Data

Policy information about availability of data

All manuscripts must include a data availability statement. This statement should provide the following information, where applicable:

- Accession codes, unique identifiers, or web links for publicly available datasets
- A description of any restrictions on data availability
- For clinical datasets or third party data, please ensure that the statement adheres to our policy

Raw sequencing data files of patients cannot be publicly shared under the obtained institutional review board approval, as patients did not consent to share raw sequencing data beyond the research and clinical terms. However, the datasets generated and analyzed during the current study are available from the corresponding author on reasonable request and upon a data usage agreement.

Human research participants

Policy information about studies involving human research participants and Sex and Gender in Research.

Reporting on sex and gender	Female patients only since we focused on ovarian cancer.
Population characteristics	Eligibility criteria included being at least 18 years old at diagnosis; a histological/pathologic diagnosis of EOC (ovarian cancer, fallopian tube cancer, or primary peritoneal cancer); high-grade serous or grade-3 endometroid histological subtype; FIGO stage II, III, or IV; first-line surgery performed; administered at least 5 rounds of first-line chemotherapy. Eligibility criteria unique to the FACT cohort included no maintenance therapy of any kind, no PARPi administered during first-line therapies for any purpose, and the date of the last dose of first-line adjuvant chemotherapy between 2009/12/01 and 2020/05/01. The FACT cohort patients were collected from two different investigators. Eligibility criteria unique to FPMT included achieving complete or partial response after first-line adjuvant chemotherapy and PARPi administered as first-line maintenance therapy.
Recruitment	Medical records of EOC patients from Peking Union Medical College Hospital and West China Hospital of Sichuan University were surveyed retrospectively.
Ethics oversight	The study was approved by the Ethics Committee of Peking Union Medical College Hospital, Beijing (project ID: JS–1932).

Note that full information on the approval of the study protocol must also be provided in the manuscript.

Field-specific reporting

Please select the one below that is the best fit for your research. If you are not sure, read the appropriate sections before making your selection.

☒ Life sciences ☐ Behavioural & social sciences ☐ Ecological, evolutionary & environmental sciences

For a reference copy of the document with all sections, see nature.com/documents/nr-reporting-summary-flat.pdf

Life sciences study design

All studies must disclose on these points even when the disclosure is negative.

Sample size	The sample size was determined to detect a sufficient difference between two groups. This is the largest sample size for such an analysis.
Data exclusions	No data were excluded
Replication	NA
Randomization	NA
Blinding	NA

Reporting for specific materials, systems and methods

We require information from authors about some types of materials, experimental systems and methods used in many studies. Here, indicate whether each material, system or method listed is relevant to your study. If you are not sure if a list item applies to your research, read the appropriate section before selecting a response.

Materials & experimental systems

n/a	Involved in the study	
☒	☐	Antibodies
☒	☐	Eukaryotic cell lines
☒	☐	Palaeontology and archaeology
☒	☐	Animals and other organisms
☐	☒	Clinical data
☒	☐	Dual use research of concern

Methods

n/a	Involved in the study	
☒	☐	ChIP-seq
☒	☐	Flow cytometry
☒	☐	MRI-based neuroimaging

Clinical data

Policy information about clinical studies

All manuscripts should comply with the ICMJE guidelines for publication of clinical research and a completed CONSORT checklist must be included with all submissions.

Clinical trial registration	NA
Study protocol	The study was approved by the Ethics Committee of Peking Union Medical College Hospital, Beijing (project ID: JS–1932).
Data collection	Medical records of EOC patients from Peking Union Medical College Hospital and West China Hospital of Sichuan University were surveyed retrospectively.
Outcomes	OS, PFS
